# Interrupted time series study on the service efficiency and cost structure of DRG payments in the GE1 group

**DOI:** 10.3389/fpubh.2025.1649042

**Published:** 2025-11-18

**Authors:** Xiaoyan Yu, Hong Zhang, Lili Shen, Juan Chen, Jian Shi, Jinhong Cai, Xinwei Chen, ChunLi Huang, Huiling Wang

**Affiliations:** 1 Medical Insurance Management Section, Haimen District People's Hospital, Nantong, China; 2 Department of Critical Care Medicine, Haimen District People's Hospital, Nantong, China; 3 Medical Affairs Management Department, Haimen District People's Hospital, Nantong, China; 4 Administrative Department, Haimen District People's Hospital, Nantong, China; 5 Medical Records Management Department, Haimen District People's Hospital, Nantong, China

**Keywords:** disease-related group (DRG), MDT, service efficiency, cost structure, interrupted time series

## Abstract

**Objective:**

Analyzing the changes in service efficiency and medical cost structure of the GE1 group (including GE13 and GE15) in a tertiary comprehensive medical institution in Nantong, China, before and after the implementation of Multi-Disciplinary Team (MDT)-based DRG payment management.

**Methods:**

Data from Nantong medical insurance patients in the GE13 and GE15 groups from the sample hospital between 2022 and 2023 were collected. The Mann-Whitney U test was used to analyze changes in medical cost structure and service efficiency indicators, and an interrupted time series (ITS) model was employed to examine the trends of these changes.

**Results:**

The median length of hospital stay for patients in the GE13 group decreased from 10 to 7 days (*P* < 0.01), and the average cost per hospitalization decreased by 4,912.89 yuan (*P* < 0.01). The median length of hospital stay for patients in the GE15 group decreased from 8 to 6 days (*P* < 0.01), and the average cost per hospitalization was reduced by 2,156.01 yuan (*P* < 0.01). The ITS analysis of post-intervention trends showed that the intervention measures for the GE13 group achieved significant results in terms of length of hospital stay, average cost per admission, and medication costs. However, some indicators, such as material costs, examination fees, and laboratory test costs, did not exhibit a significant downward trend (*P* > 0.05).

**Conclusion:**

After implementing MDT-based DRG management, the operational efficiency of the GE13 and GE15 groups improved, physicians' diagnostic and treatment behaviors became more standardized, medical quality was enhanced, medical costs were controlled, patients' hospitalization burden was reduced, and the issue of hospital medical insurance settlement losses was reversed. It is recommended to continue implementing MDT management for DRG groups, continuously refine pathways, optimize structures, improve efficiency, and strengthen medical record supervision. This study was conducted in the context of China's DRG reform and directly demonstrates the effectiveness of the reform in controlling healthcare costs. It is important to note that cost control was not achieved by reducing medical services or shifting patients elsewhere, but rather by improving resource utilization efficiency while ensuring the quality of care. This research reinforces the credibility of the DRG reform policy and provides a basis for higher-level healthcare insurance authorities to adjust diagnosis-related group payment standards and coordinate supporting policies.

## 1 Introduction

Diagnosis-related groups (DRGs) are widely regarded as one of the most advanced payment systems in both academic and public perception worldwide (1). DRG payment is of great significance in achieving a win-win situation for “hospitals, insurers, and patients,” promoting tiered healthcare delivery, and facilitating the transformation of service models. In November 2021, China's National Healthcare Security Administration issued the *Three-Year Action Plan for DRG/DIP Payment Reform*, requiring that DRG/DIP payment methods cover all eligible medical institutions providing inpatient services by the end of 2025 (2). This move officially commenced the reform of the DRG payment method. In September 2022, the Nantong Municipal Healthcare Security Administration issued the *Notice on Implementing DRG Point-Based Payment and Settlement in Nantong* (3), proposing that the city would formally implement DRG point-based payment and settlement starting from 2022.

According to policy requirements, Nantong Haimen People's Hospital (hereinafter referred to as the sample hospital) implemented DRG-based medical insurance settlement in 2022. The settlement results showed that the GE13 and GE15 diagnosis groups were the most loss-making conditions in the general surgery department, exhibiting issues of cost overruns and inefficiency. Inguinal and ventral hernia surgeries are basic and common procedures in general surgery, making the analysis of the GE13 and GE15 groups representative and typical. From the perspective of improving internal management, the hospital established a multi-disciplinary team (MDT) for DRG management. The MDT is composed of departments such as Healthcare Insurance Management, Medical Affairs, Operations Management Office, and Pharmacy Starting from January 2023, measures such as operational analysis, clinical pathways, and performance evaluation were implemented to enhance quality and efficiency. This study collected data from Nantong medical insurance patients in the GE13 and GE15 groups at the sample hospital between 2022 and 2023. An interrupted time series (ITS) model was used to evaluate changes in service efficiency and cost structure for the GE13 and GE15 groups, summarizing practical experiences in DRG payment management.

## 2 Materials and methods

### 2.1 Data sources

The study selected cases from Nantong medical insurance patients discharged between 2022 and 2023 at the sample hospital that were grouped into the GE13 and GE15 categories according to the China Healthcare Security Diagnosis Related Groups (CHS-DRG) grouping system as the research subjects. Cases involving fragmented hospitalization—i.e., readmission within 15 days after discharge into the same DRG group—were excluded, as were cases involving simultaneous bilateral inguinal hernia surgery, to minimize the impact of special circumstances on the data. The data were sourced from the Nantong medical insurance settlement system, including grouping results and detailed cost records, ensuring authenticity, reliability, and representativeness.

### 2.2 Indicator determination

According to the principles and characteristics of the CHS-DRG grouping system, cases within the same DRG group exhibit similar treatment processes and resource consumption. Therefore, indicators such as average length of stay (ALOS), average cost per hospitalization, Time Consumption Index, and Cost Consumption Index were selected as metrics for evaluating service efficiency. Among these, the Time Consumption Index and Cost Consumption Index are indicators used to measure the time and cost required to treat the same category of disease. The Time Consumption Index is calculated as the average length of stay for a specific DRG group divided by the regional average length of stay for that same DRG group. Similarly, the Cost Consumption Index is calculated as the average cost for a specific DRG group divided by the regional average cost for that same DRG group (4). When the index value is 1, it indicates that the time or cost consumption for treating the same category of disease is equivalent to the average level of hospitals across the city (5). The average cost per hospitalization, as well as itemized costs such as average medication and medical supply expenses, were used as evaluation metrics for the cost structure (6).

### 2.3 Research methods

January 2023 was designated as the intervention point for the implementation of management measures, with the year 2022 representing the pre-intervention phase and 2023 the post-intervention phase. Referring to the cost classification of the Nantong Medical Insurance Settlement System, relevant costs were categorized according to medications, medical supplies, examinations, laboratory tests, and medical services.

Interrupted time series (ITS) analysis does not rely on a control group. By precisely analyzing the dynamic effects of interventions along the “time dimension,” it can finely capture both the “immediate effects” and “long-term trend effects” of an intervention. In contrast, other quasi-experimental methods tend to focus more on “average effects” and struggle to reflect the temporal dynamics of these effects. Therefore, we selected ITS for our analysis.

As the hospitalization costs for DRG groups do not follow a normal distribution, continuous variables were expressed as medians and interquartile ranges (M [P25, P75]). SPSS 26.0 software was used to perform the Mann–Whitney *U* test on the hospitalization costs of the GE13 and GE15 groups for the years 2022 and 2023. The research hypothesis focused on whether differences existed in the GE13 group before and after the implementation of MDT-based group management, using a two-tailed test. A *P*-value < 0.05 was considered statistically significant.

Using STATA 14.0, observation points were marked monthly based on discharge dates (totaling 24 months), and an interrupted time series (ITS) model was established to observe the changing trends in hospitalization costs and their structure. The equation was as follows: Yt = β0 + β1 time + β2 intervention + β3 trend + ϵt. Where:β0 is the constant term (intercept), β1 is the slope of the outcome variable before the intervention (baseline time trend), β_2_ represents the change in the level of the outcome variable immediately after the intervention (instantaneous intervention effect), β3 indicates the change in slope after the intervention (long-term effect over time), and β1 + β3 represents the post-intervention trend.

Since the observational data in this study were all obtained from normally grouped data feedback provided by the Medical Insurance Bureau (MIB), there were no missing data.

## 3 Results

### 3.1 Baseline data for the GE13 and GE15 groups

In 2022, the sample hospital grouped 65 cases into GE13, with an average cost per case of ¥18,657.20 and an average loss per case of -¥3,107.18. For GE15, 220 cases were grouped, with an average cost per case of ¥14,260.51 and an average loss per case of -¥1,879.30. Both diagnosis groups showed significant financial losses.

The financial losses in the GE13 group are not an isolated case but rather a systemic and structural issue, where actual resource consumption exceeds the DRG payment standards, affecting the overall operational efficiency of the hospital. The data serves as a strong warning signal, indicating that there is room for optimization in areas such as medical supply usage, average length of stay, and clinical pathway management for patients in this diagnosis group. This drives the hospital to implement refined management reforms, shifting from extensive growth to intensive development.

Simultaneously, the analysis results of loss-making cases are more intuitively fed back to clinical physicians, enabling them to clearly see the gap between their diagnostic and treatment behaviors and the DRG payment standards. This guides doctors to adopt a cost-effectiveness mindset while ensuring medical quality and safety, proactively opting for treatment plans with higher cost-effectiveness.

### 3.2 Changes in service efficiency and cost structure

#### 3.2.1 Service efficiency metrics

In 2023, a total of 385 cases of inguinal and ventral hernia surgeries were grouped at the sample hospital, with 117 cases in the GE13 group and 268 cases in the GE15 group. Analysis using the interrupted time series model revealed, as shown in Tables 1, 2, that compared to 2022, the average length of stay, average cost per case, time consumption index, and cost consumption index for both the GE13 and GE15 groups decreased in 2023 (*P* < 0.01). The improvement in these metrics reflects a significant enhancement in service efficiency following the implementation of management measures.

**Table 1:** Changes and comparative analysis of each indicator for group GE13 from 2022–2023.

	**Time median M (P25, P75)**		***Z***	***P-*value**
	**2022 year (*****n*** = **65)**	**2023 year (*****n*** = **117)**		
Length of stay	10.00 (8.00, 13.50)	7.00 (5.00, 9.00)	−5.715	0.000^**^
Average cost per visit	16,637.28 (13,301.56, 19,485.52)	11,724.39 (9,535.87, 14,473.60)	−5.434	< 0.001^**^
Time consumption index	1.10 (0.88, 1.48)	0.77 (0.55, 0.99)	−5.902	< 0.001^**^
Cost consumption index	1.17 (0.94, 1.37)	0.83 (0.67, 1.02)	−5.439	< 0.001^**^

^*^P < 0.05; ^**^P < 0.01.

**Table 2:** Changes and comparative analysis of each indicator for group GE15 from 2022–2023.

	**Time median M (P**_**25**_ **, P**_**75**_ **)**		***Z***	***P-*value**
	**2022 year (*****n*** = **220)**	**2023 year (*****n*** = **268)**		
Length of stay	8.00 (6.00, 9.00)	6.00 (5.00, 8.00)	−7.429	0.000^**^
Average cost per visit	13,741.02 (11,648.80, 16,255.76)	11,585.01 (9,731.97, 13,475.27)	−7.814	< 0.001^**^
Time consumption index	1.12 (0.91, 1.36)	0.91 (0.76, 1.21)	−7.429	< 0.001^**^
Cost consumption index	1.19 (1.01, 1.40)	1.00 (0.84, 1.17)	−7.818	< 0.001^**^

^*^P < 0.05; ^**^P < 0.01.

#### 3.2.2 Hospital costs and their structure

As shown in Tables 3, 4, significant changes were observed in various cost indicators for the GE13 and GE15 groups between 2022 and 2023. Both groups demonstrated a notable reduction in the average total cost per case (*P* < 0.01), indicating positive achievements in the hospital's cost control efforts. The median drug costs decreased significantly, which may be attributed to optimized medication management practices, such as prioritizing the use of centrally procured drugs, stringent management of antibiotics, auxiliary medications, and proton pump inhibitors, as well as restrictions on the types and dosages of discharge medications. These changes reflect improvements in treatment protocols or enhanced efficiency in drug utilization. The GE15 group showed a slight reduction in material costs, though the change was not statistically significant (*P* = 0.143), suggesting that further attention is needed in this area. Both examination and laboratory test costs exhibited significant declines, demonstrating effective cost control in these areas. The pronounced downward trend in service costs reflects an overall reduction in healthcare service expenses.

**Table 3:** Changes and comparative analysis of the cost indicators for the GE13 group from 2022–2023 (cost unit: yuan).

	**Time median M (P**_**25**_ **, P**_**75**_ **)**		***Z***	***P-*value**
	**2022 year (*****n*** = **65)**	**2023 year (*****n*** = **117)**		
Total costs	16,637.28 (13,301.56, 19,485.52)	11,724.39 (9,535.87, 14,473.60)	−5.434	< 0.001^**^
Average drug costs per case	3,445.19 (2,286.24, 5,202.66)	1,518.44 (829.19, 2,372.30)	−6.814	< 0.001^**^
Average consumable costs per case	4,638.96 (3,350.27, 5,495.22)	3,624.92 (2,426.64, 4,583.85)	−2.682	0.007^**^
Average examination costs per case	1,164.00 (948.20, 1,799.70)	993.00 (805.90, 1,362.22)	−2.174	0.030^*^
Average test costs per case	1,987.50 (1,352.25, 2,450.75)	1,383.50 (1,168.50, 1,873.75)	−3.591	< 0.001^**^
Average service costs per case	5,037.90 (4,244.40, 5,972.30)	3,935.00 (3,405.70, 5,105.15)	−4.468	< 0.001^**^

^*^P < 0.05; ^**^P < 0.01.

**Table 4:** Changes and comparative analysis of the cost indicators for the GE15 group from 2022–2023 (cost unit: yuan).

	**Time median M (P**_**25**_ **, P**_**75**_ **)**		***Z***	***P-*value**
	**2022 year (*****n*** = **220)**	**2023 year (*****n*** = **268)**		
Total costs	13,741.02 (11,648.80, 16,255.76)	11,585.01 (9,731.97, 13,475.27)	−7.814	< 0.001^**^
Average drug costs per case	2,017.45 (1,423.77, 3,260.26)	1,175.84 (745.04, 2,054.30)	−8.575	< 0.001^**^
Average consumable costs per case	3,783.86 (3,307.61, 4,830.10)	3,747.78 (2,982.03, 4,543.86)	−1.463	0.143
Average examination costs per case	993.20 (845.10, 1,231.20)	879.90 (686.40, 1,077.90)	−5.416	< 0.001^**^
Average test costs per case	1,564.25 (1,153.00, 2,099.88)	1,222.50 (1,003.13, 1,529.38)	−6.355	< 0.001^**^
Average service costs per case	4,932.00 (4,092.70, 5,524.13)	4,301.00 (3,593.00, 4,930.08)	−6.819	< 0.001^**^

^*^P < 0.05; ^**^P < 0.01.

With the exception of material costs in the GE15 group, all other indicators showed *P*-values less than 0.05, highlighting improvements in hospital management and medical efficiency. The implementation of intervention measures has further reduced operational costs, standardized clinical practices, decreased DRG-related financial losses, and effectively enhanced the patient experience by alleviating financial burdens and increasing patient satisfaction.

### 3.3 Interrupted time series analysis

#### 3.3.1 The interrupted time series graphs for the GE13 and GE15 groups

As visually illustrated in Figures 1–9, the trends of various indicators for the GE13 group following the implementation of the intervention measures can be intuitively observed.

**Figure 1:**
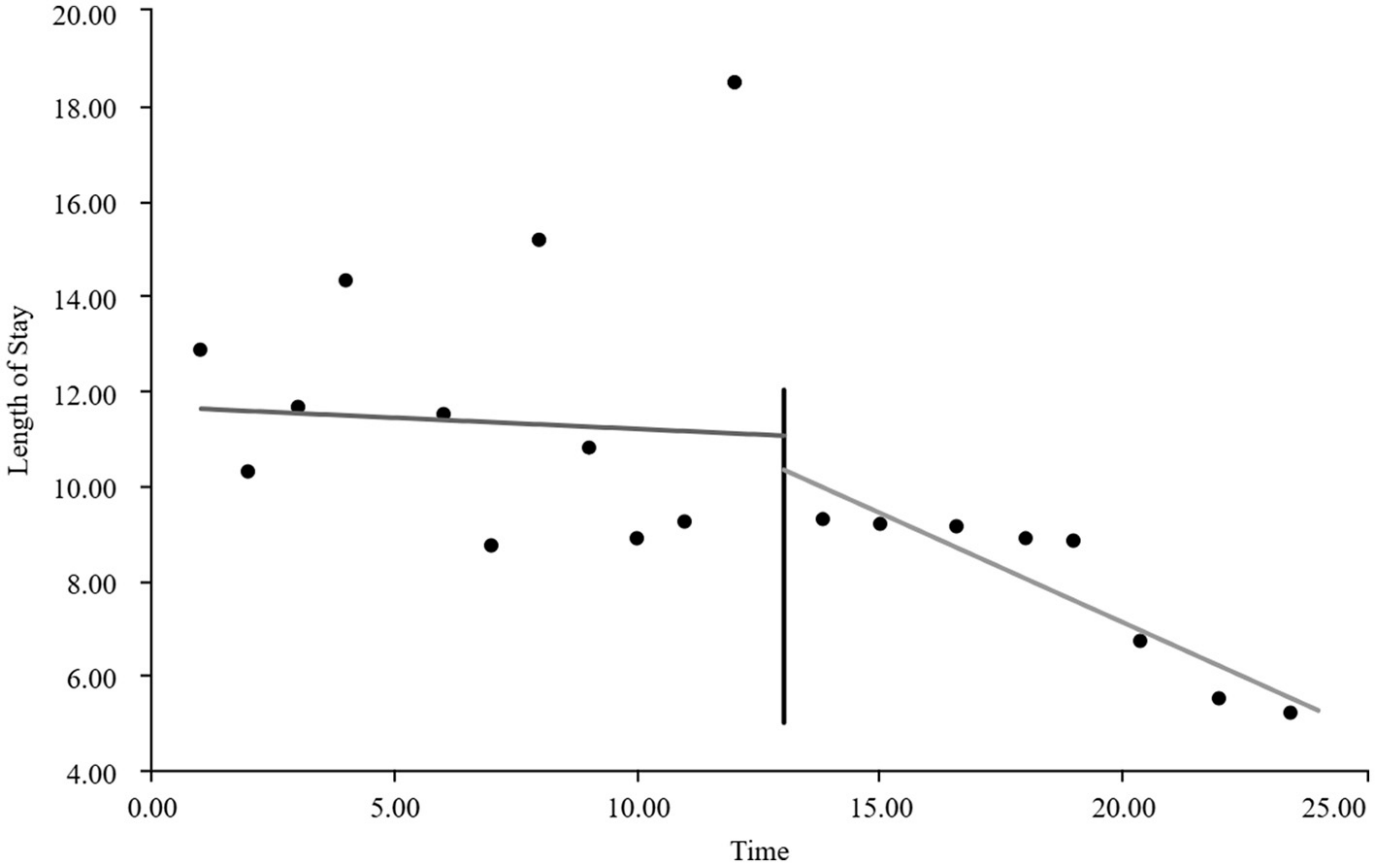
Changes in the average length of hospital stay for the GE13 group.

**Figure 2:**
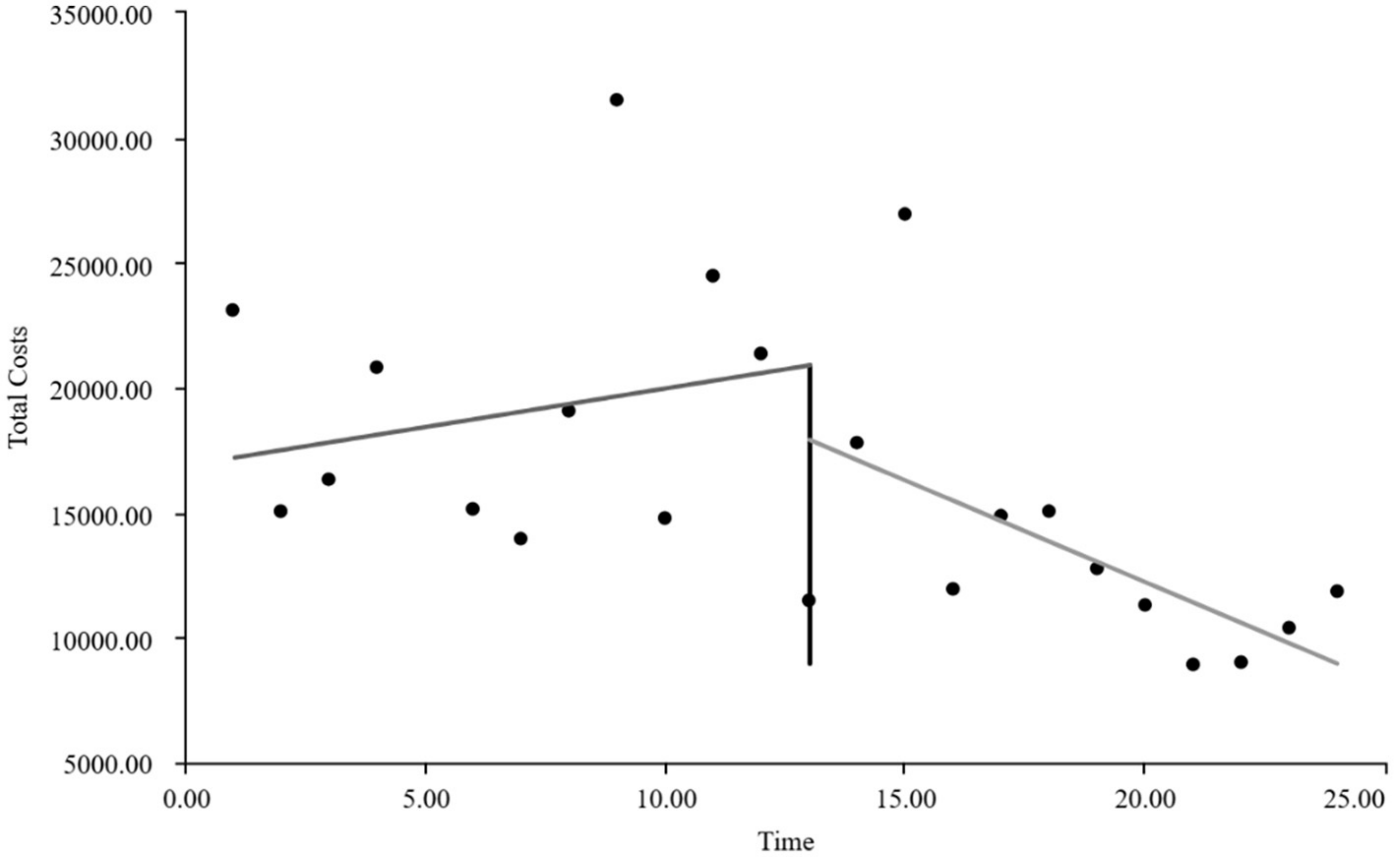
Changes in average total costs per case for the GE13 group.

**Figure 3:**
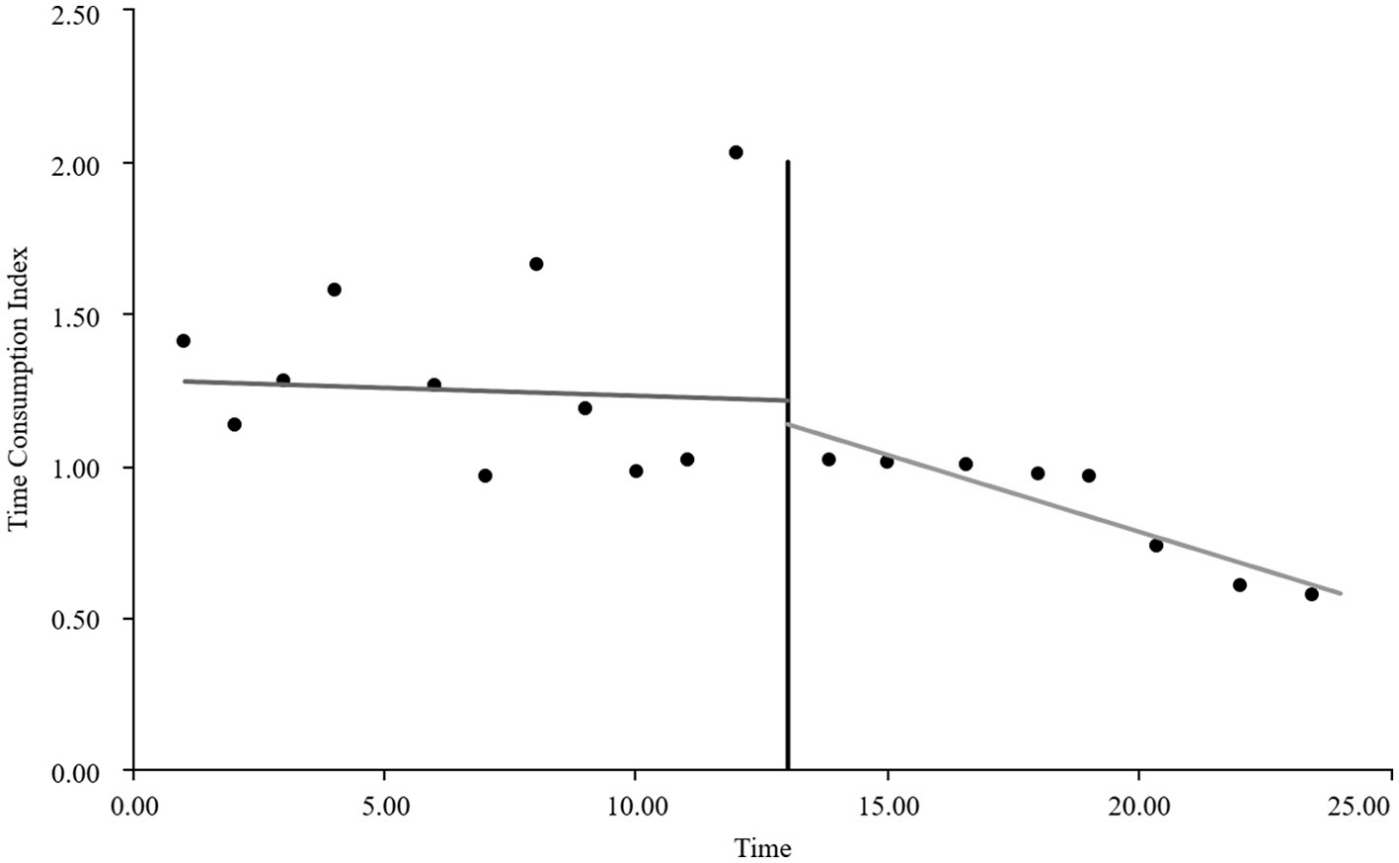
Changes in time consumption index for the GE13 group.

**Figure 4:**
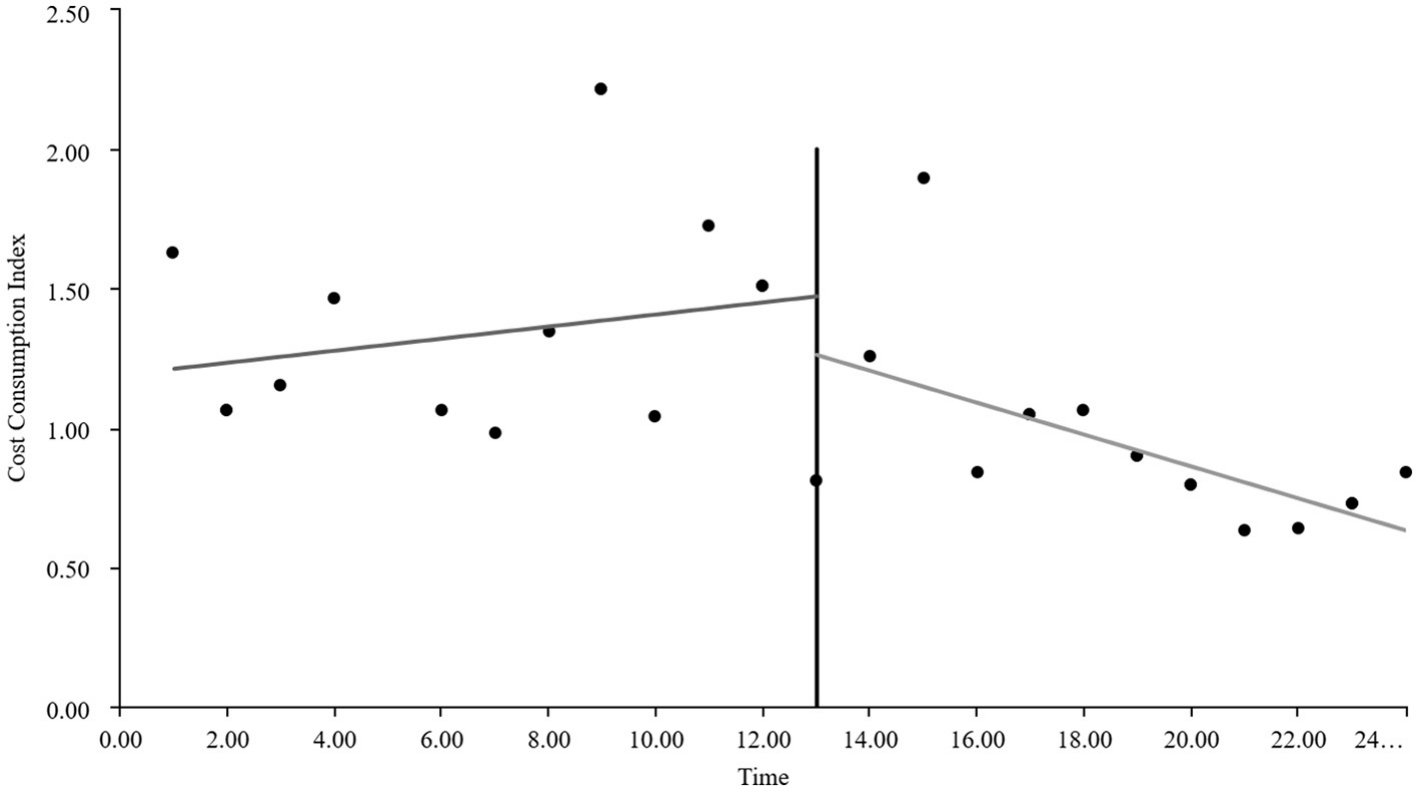
Changes in cost consumption index for the GE13 group.

**Figure 5:**
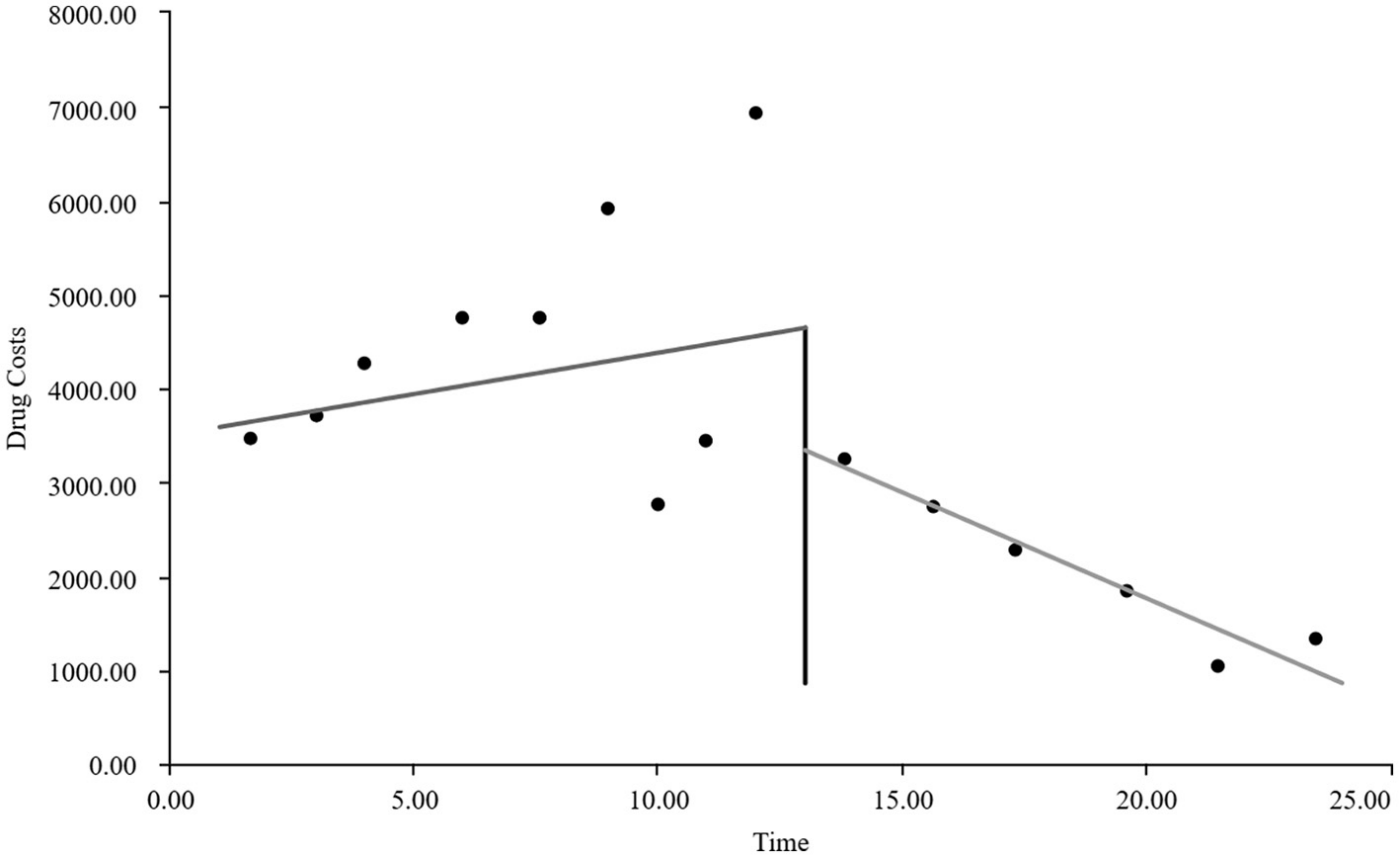
Changes in average drug costs per admission for the GE13 group.

**Figure 6:**
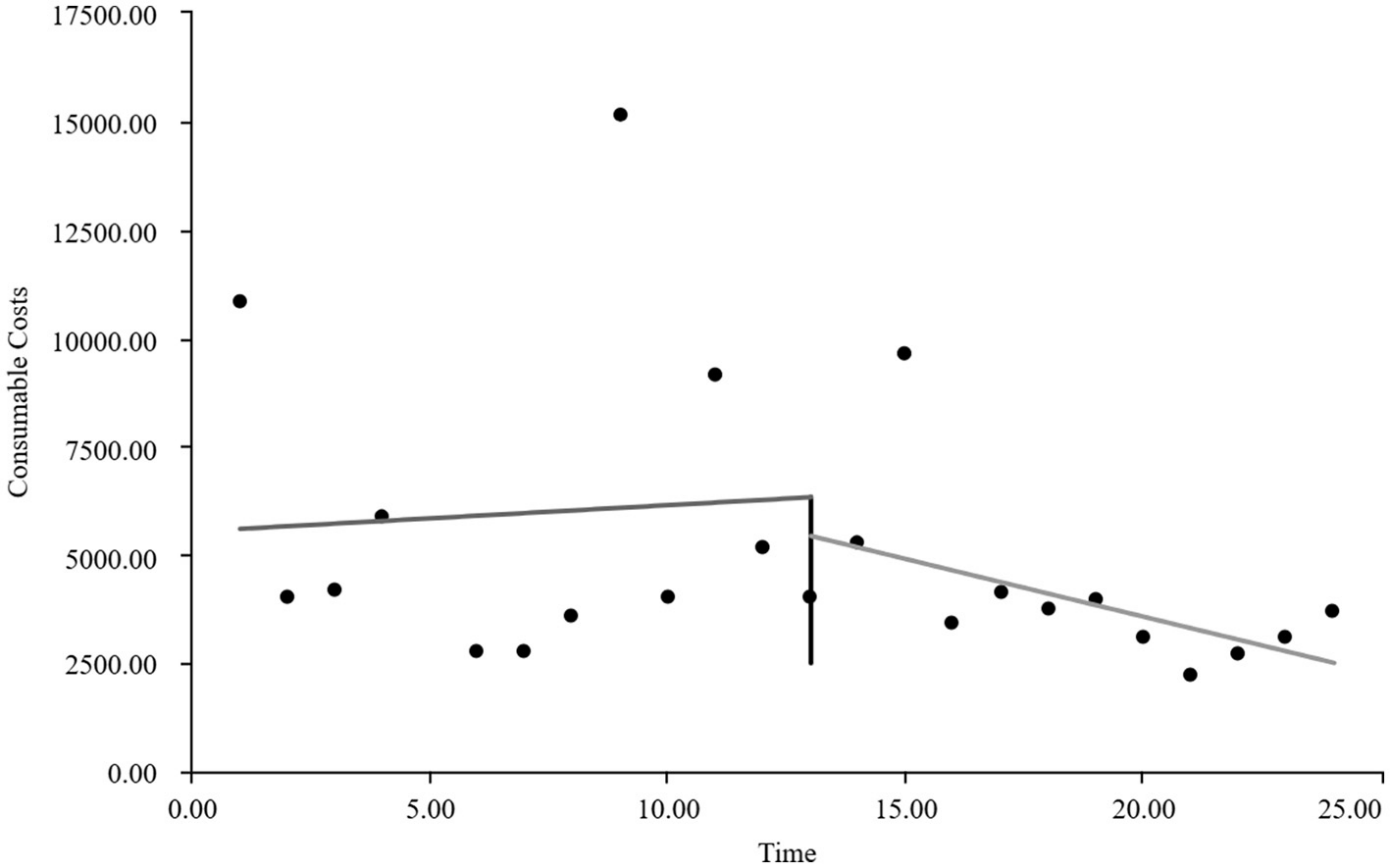
Changes in average consumables costs per visit for the GE13 group.

**Figure 7:**
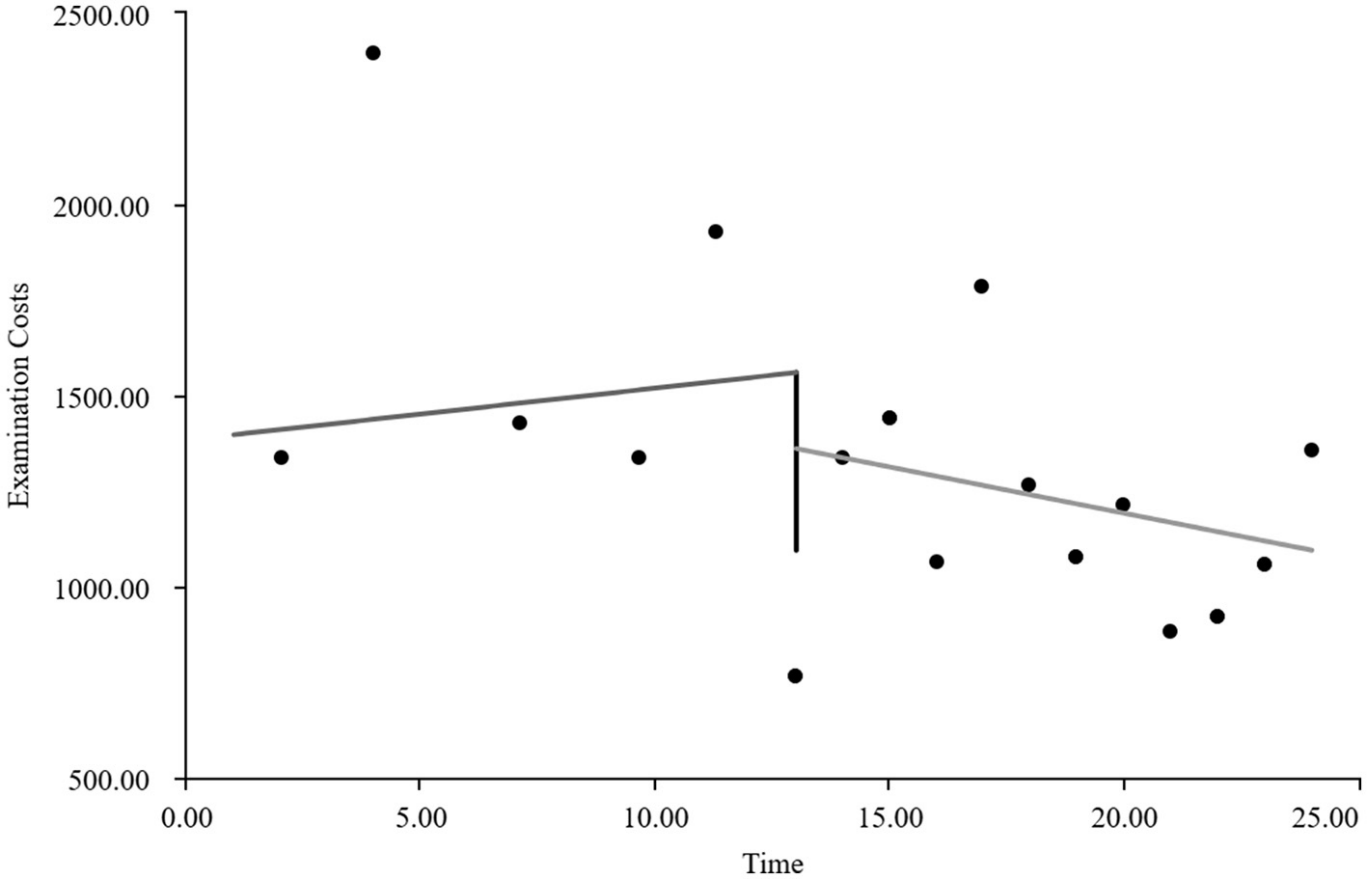
Changes in average examination costs per group for GE13.

**Figure 8:**
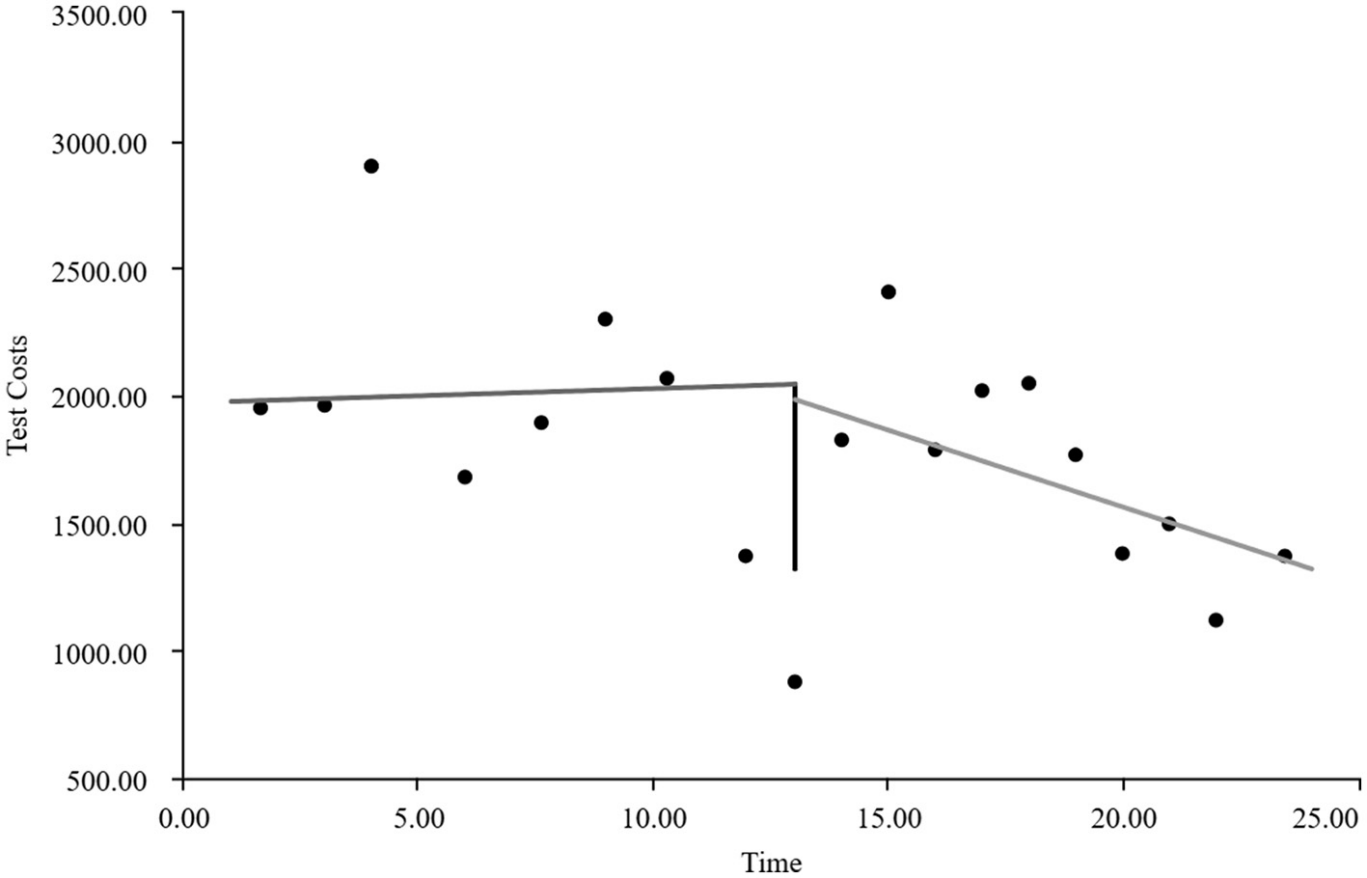
Changes in average test costs per session in group GE13.

**Figure 9:**
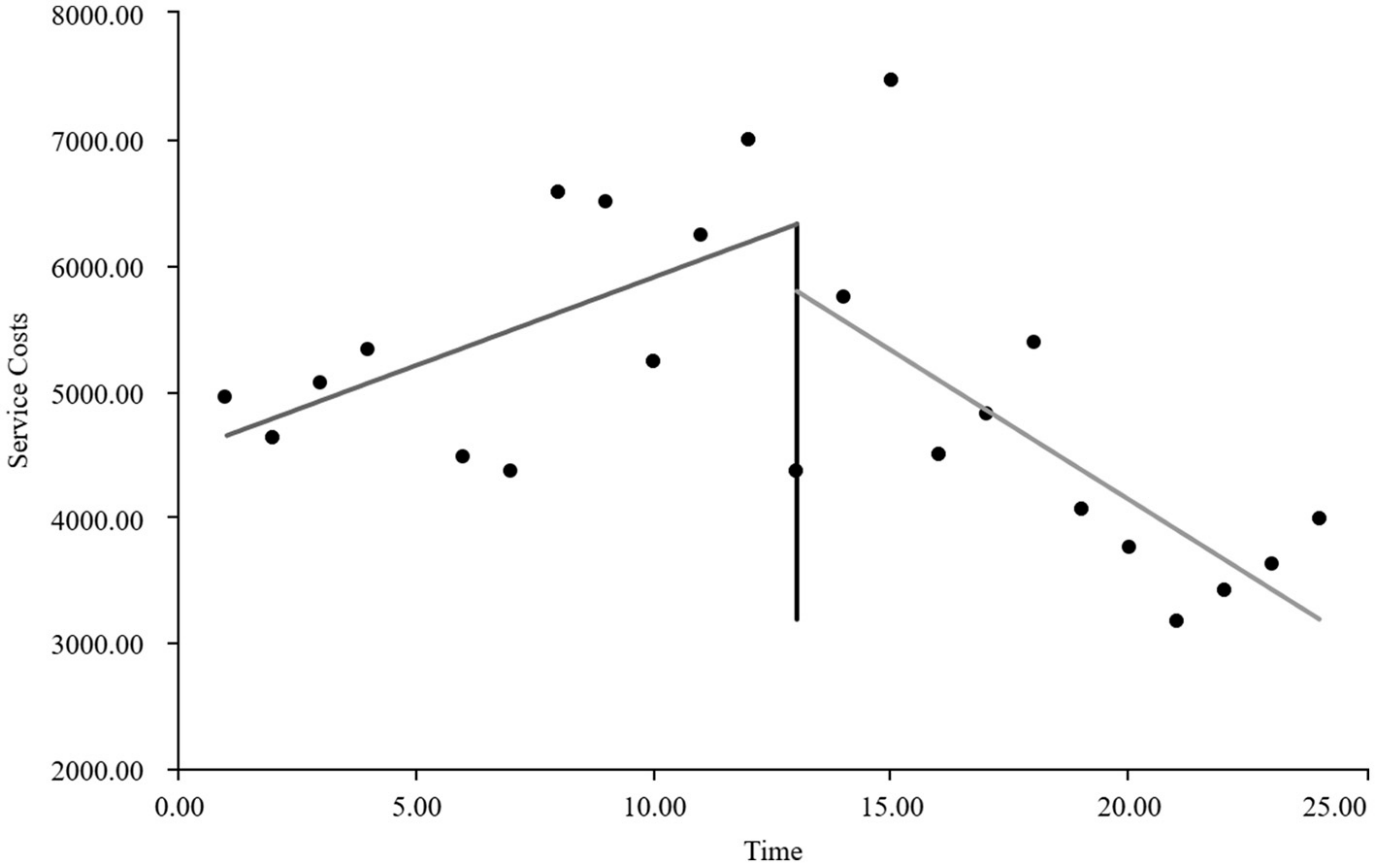
Changes in average service costs for consumables in group GE13 per Session.

As can be intuitively observed from Figures 10–18, the trends of various indicators for the GE15 group after the implementation of the intervention measures are clearly demonstrated.

**Figure 10:**
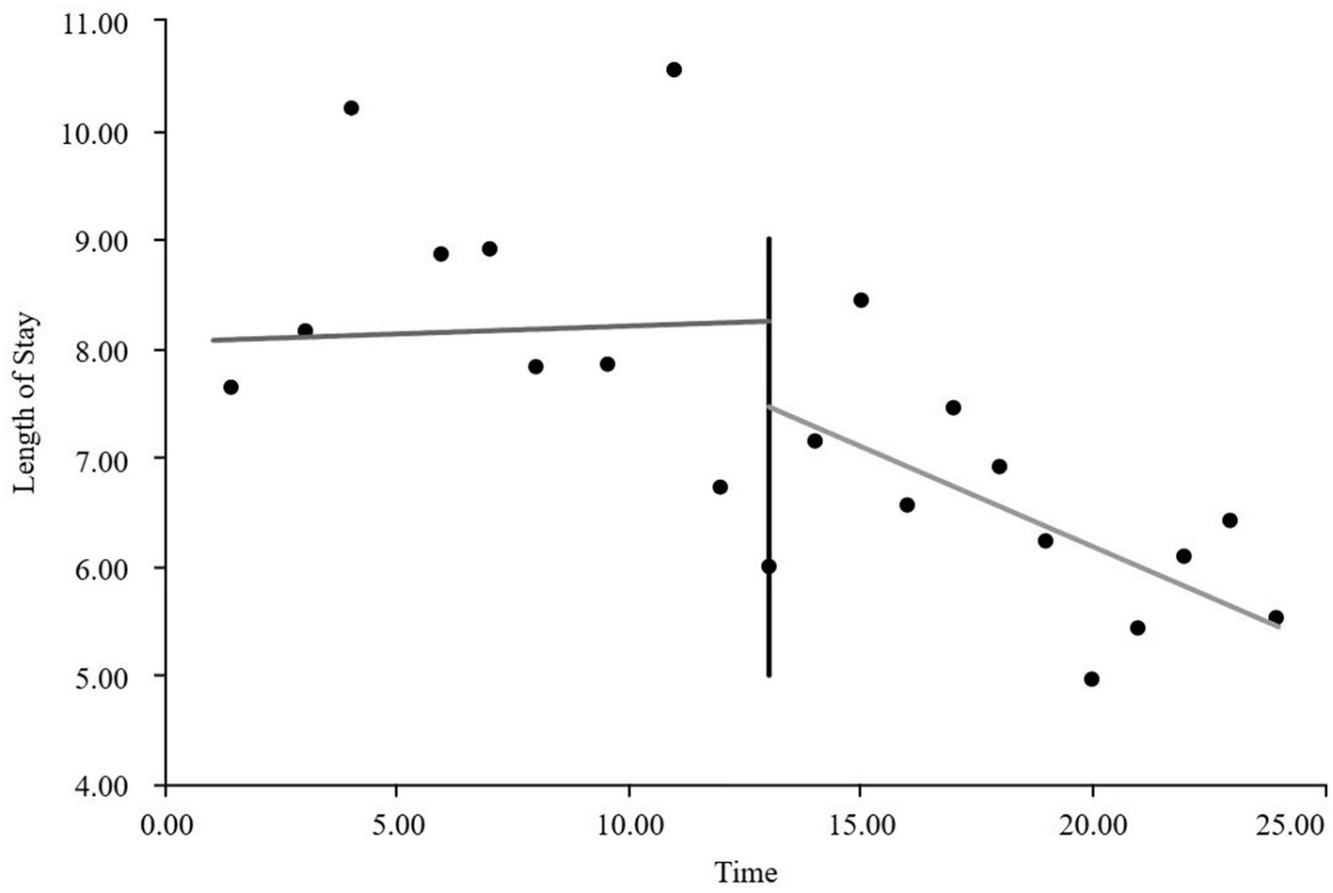
Trends of changes in average length of hospital stay for group GE15.

**Figure 11:**
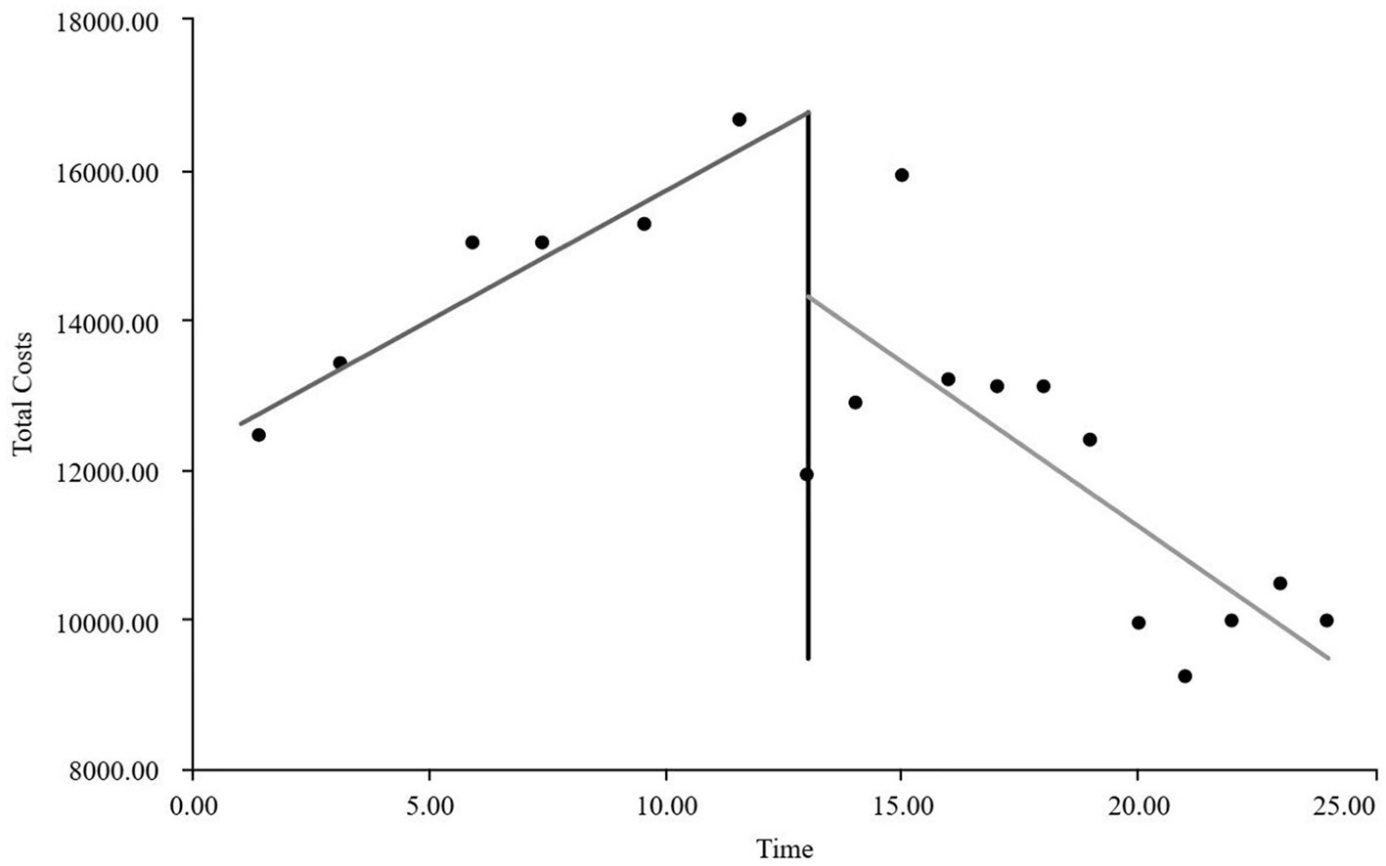
Trends of changes in average hospitalization costs per session for group GE15.

**Figure 12:**
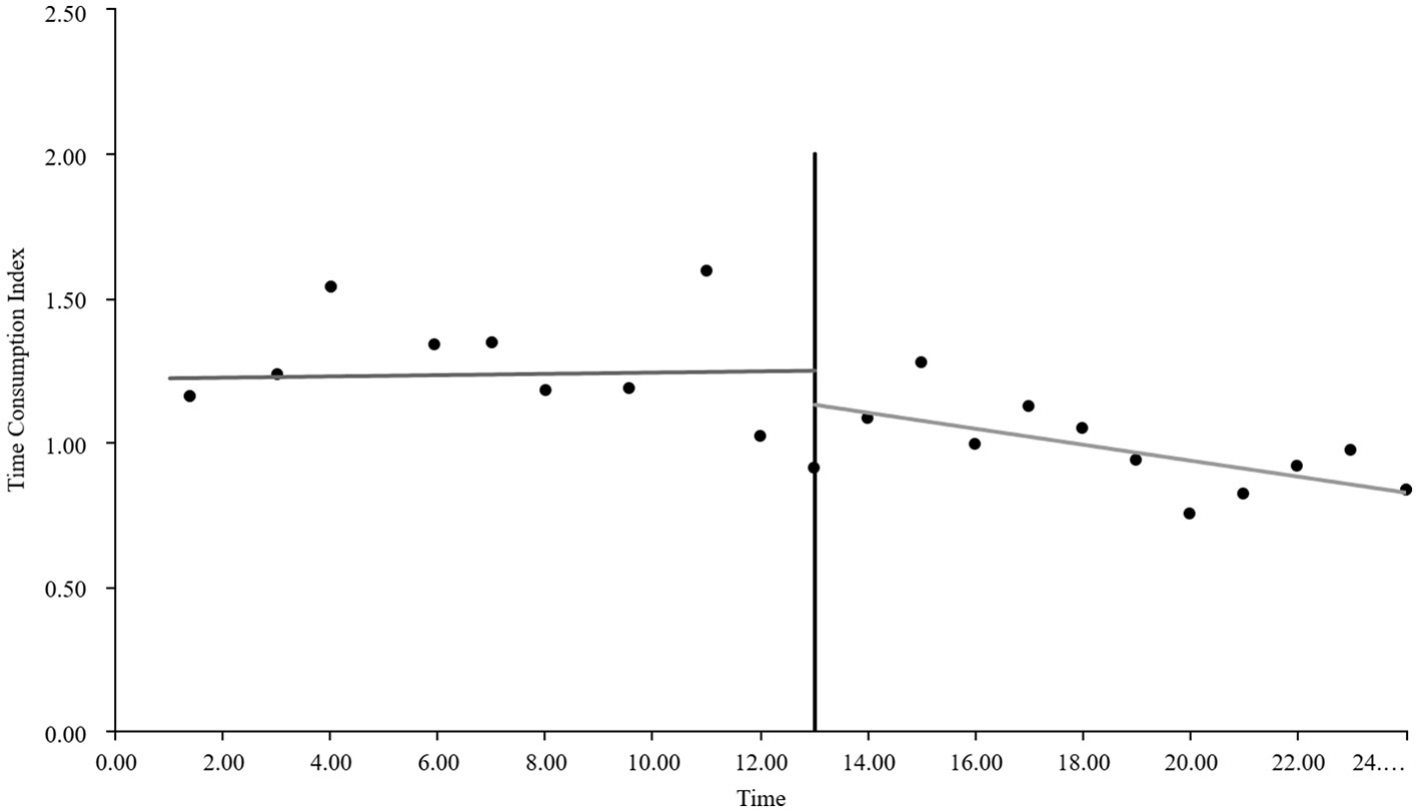
Changes in time consumption index for the GE15 group.

**Figure 13:**
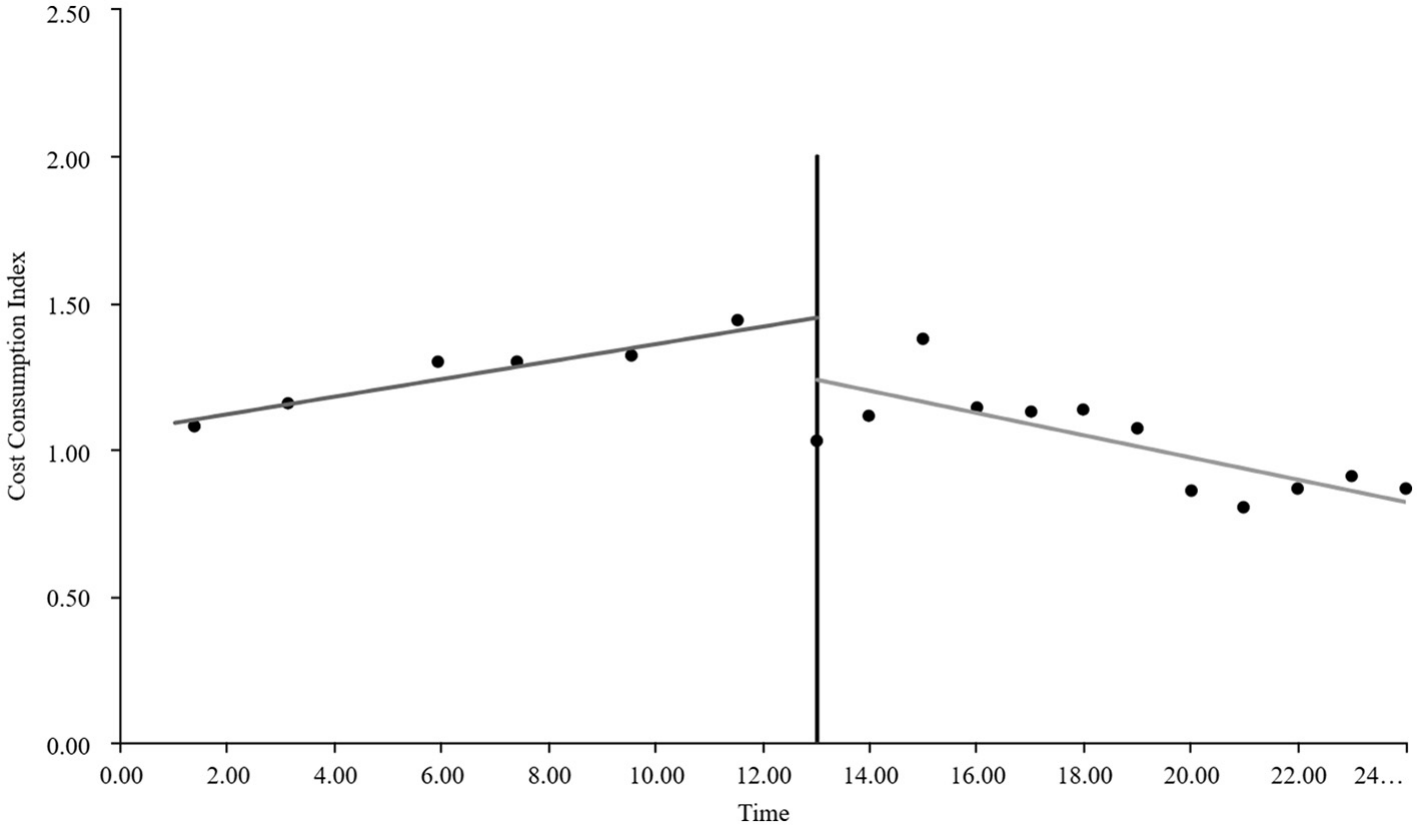
Changes in cost consumption index for the GE15 group.

**Figure 14:**
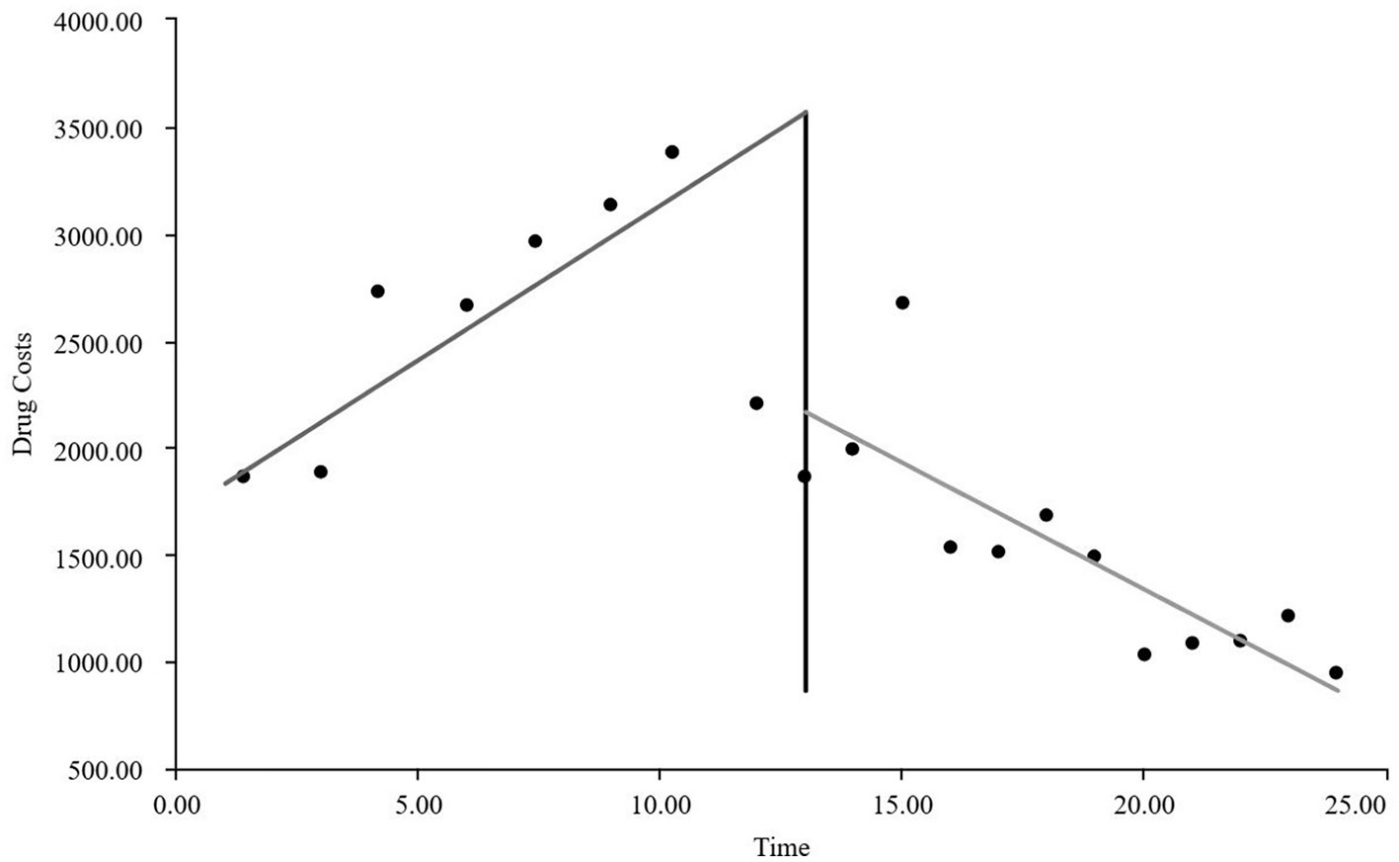
Trends of changes in average drug costs per session for group GE15.

**Figure 15:**
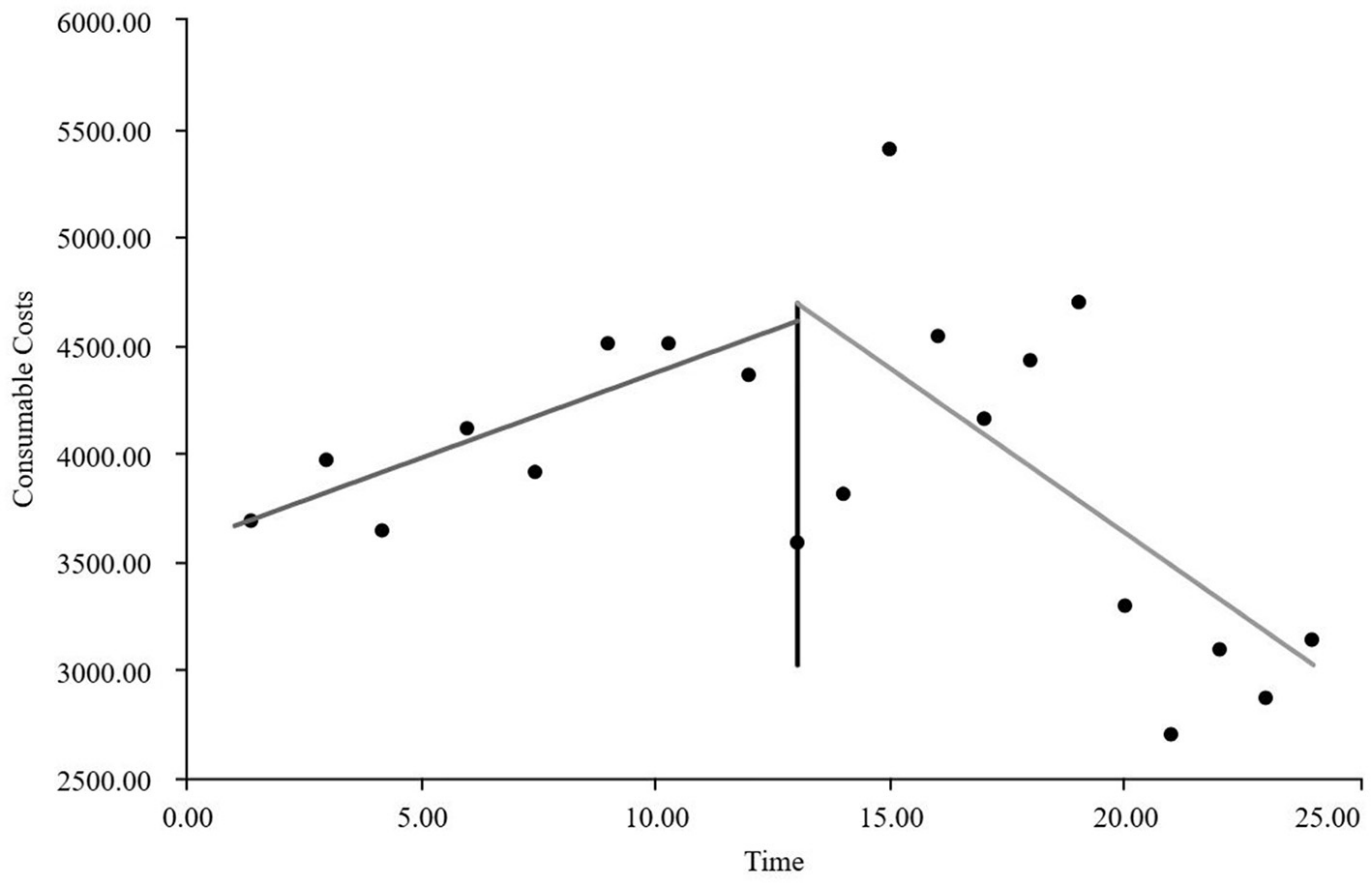
Trends in average consumables costs per visit for the GE15 group.

**Figure 16:**
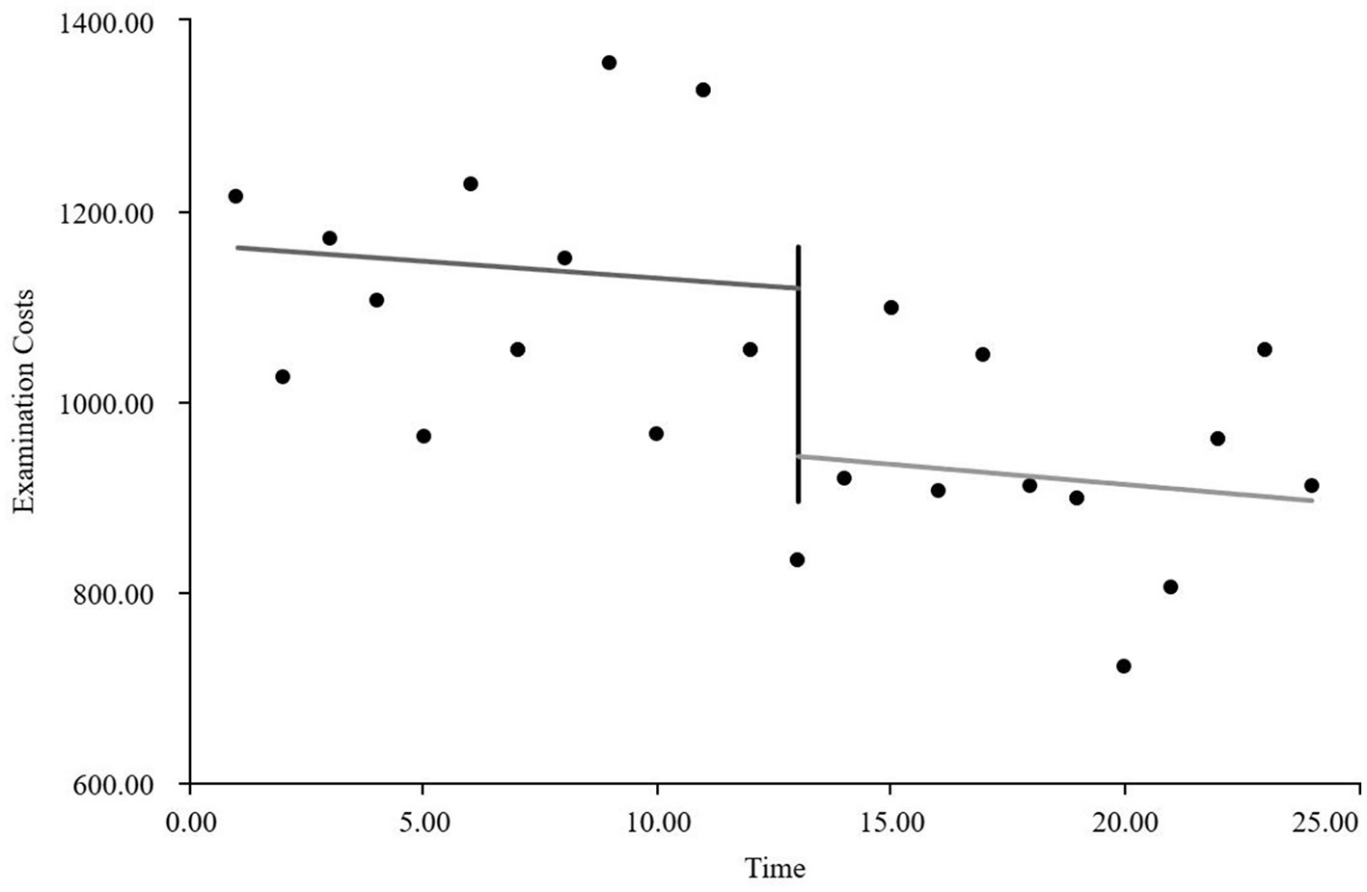
Trends of changes in average examination costs per session for group GE15.

**Figure 17:**
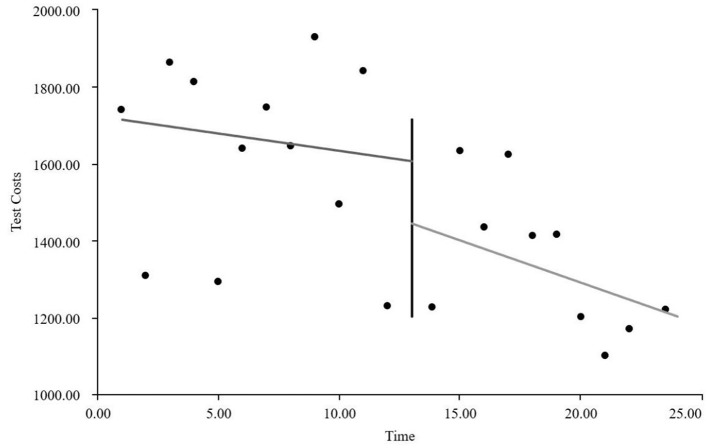
Trends of changes in average test costs per session for group GE15.

**Figure 18:**
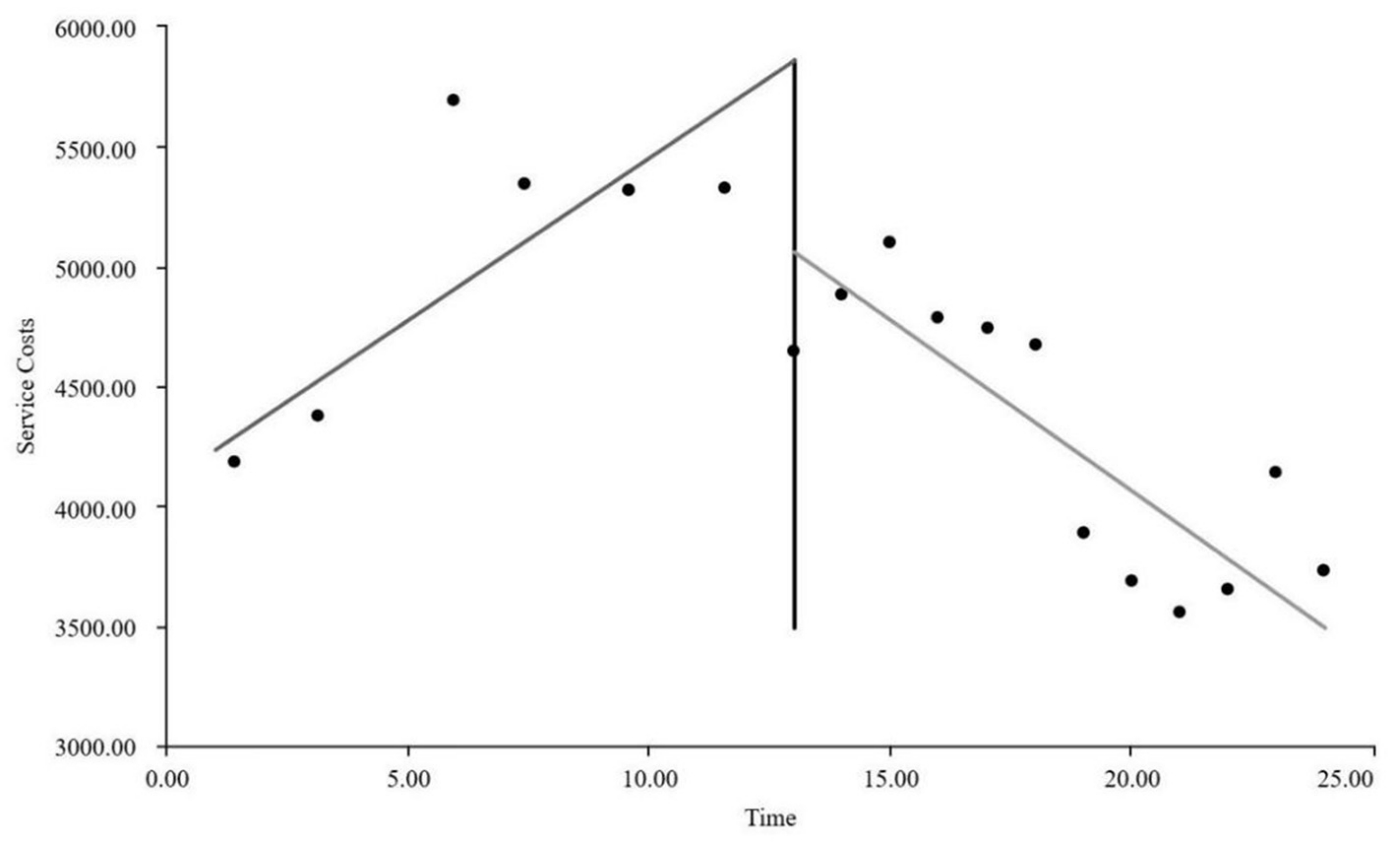
Trends of changes in average service costs per session for group GE15.

#### 3.3.2 Interrupted time series analysis results of various indicators for GE13 and GE15 groups

Tables 5, 6 present the interrupted time series analysis results of various indicators for the GE13 and GE15 groups, including model parameter estimates for length of stay, total cost, time efficiency index, cost efficiency index, drug cost, material cost, examination fee, laboratory test cost, and service fee.

**Table 5:** Interrupted time series analysis of indicators for group GE13.

	**Length of stay**	**Total costs**	**Time consumption index**	**Cost consumption index**	**Drug costs**	**Consumable costs**	**Examination costs**	**Test costs**	**Service costs**
β_0_	11.654 (< 0.001)^**^	16,894.700 (< 0.001)^**^	1.282 (< 0.001)^**^	1.190 (< 0.001)^**^	3,499.056 (< 0.001)^**^	5,537.118 (< 0.001)^**^	1,383.801 (< 0.001)^**^	1,971.193 (< 0.001)^**^	4,503.533 (< 0.001)^**^
β_1_	−0.047 (0.732)	307.964 (0.301)	−0.005 (0.730)	0.022 (0.301)	88.084 (0.221)	60.793 (0.752)	13.544 (0.636)	5.636 (0.849)	139.907 (0.008)^**^
β_2_	−0.719 (0.594)	−2,988.862 (0.3061)	−0.078 (0.598)	−0.211 (0.306)	−1,304.4 (0.065)	−893.295 (0.636)	−198.965 (0.479)	−59.958 (0.8371)	−532.244 (0.298)
β_3_	−0.414 (0.022)^*^	−1,120.830 (0.004)^**^	−0.045 (0.022) ^*^	−0.079 (0.004)^*^	−313.395 (0.001)^**^	−327.03 (0.193)	−37.708 (0.313)	−66.032 (0.089)	−376.665 (< 0.001)^**^

The value in parentheses is the P value, ^*^P < 0.05 ^**^P < 0.01, β1 represents the pre-intervention trend, β_2_ represents the immediatie post-intervention effcctpre, β1+β3 represents the post-intervention trend.

**Table 6:** Interrupted time series analysis of indicators for group GE15.

	**Length of hospital stay**	**Total costs**	**Time consumption index**	**Cost consumption index**	**Drug costs**	**Consumable costs**	**Examination costs**	**Test costs**	**Service costs**
β_0_	8.057 (< 0.001) ^**^	12,260.614 (< 0.001) ^**^	1.129 (< 0.001) ^**^	1.061 (< 0.001) ^**^	1,687.328 (< 0.001) ^**^	3,586.231 (< 0.001) ^**^	1,164.779 (< 0.001) ^**^	1,723.354 (< 0.001) ^**^	4,098.922 (< 0.001) ^**^
β_1_	0.015 (0.754)	346.166 (< 0.001) ^**^	0.002 (0.754)	0.030 (< 0.001) ^**^	144.492 (< 0.001) ^**^	78.814 (0.012)^*^	−3.54 (0.663)	−9.001 (0.377)	135.401 (< 0.001) ^**^
β_2_	−0.786 (0.106)	−2,457.823 (< 0.001) ^**^	−0.118 (0.107)	−0.212 (< 0.001) ^**^	−1,400.017 (< 0.001) ^**^	80.902 (0.804)	−176.419 (0.038)^*^	−162.341 (0.127)	−799.948 (< 0.001) ^**^
β_3_	−0.198 (0.003) ^**^	−784.954 (< 0.001) ^**^	−0.030 (0.003) ^**^	−0.068 (< 0.001) ^**^	−262.817 (< 0.001) ^**^	−230.548 (< 0.001) ^**^	−0.693 (0.952)	−12.989 (0.372)	−277.908 (< 0.001) ^**^

The value in parentheses is the P value, ^*^P < 0.05 ^**^P < 0.01, β1 represents the pre-intervention trend, β_2_ represents the immediate post-intervention effect, β1+β3 represents the post-intervention trend.

According to Table 5, the pre-intervention trend (β1) of the GE13 group showed no significant upward or downward trend in length of stay, total cost, time efficiency index, cost efficiency index, drug cost, material cost, examination fee, or laboratory test cost (*P* > 0.05). However, service fees demonstrated a significant increasing trend before the intervention (*P* < 0.01), rising by an average of 139.907 yuan per month. The instantaneous intervention effect (β_2_) analysis indicated a decline in all indicators in the month of intervention, though these changes were not statistically significant (*P* > 0.05). The post-intervention trend (β1 + β3) revealed significant downward trends in length of stay, total cost, drug cost, service fee, time efficiency index, and cost efficiency index (*P* < 0.05), with monthly decreases of 0.461 days, 812.866 yuan, 225.311 yuan, 236.758 yuan, 0.02020, and 0.057, respectively, indicating a positive long-term impact of the interventions. However, no significant downward trends were observed in material cost, examination fee, or laboratory test cost after the intervention (*P* > 0.05), suggesting that management of these areas requires further attention.

As shown in Table 6, the pre-intervention trend (β1) of the GE15 group exhibited no significant trend in length of stay, time efficiency index, examination fee, or laboratory test cost (*P* > 0.05). In contrast, significant increasing trends were observed in total cost, drug cost, material cost, service fee, and cost efficiency index (*P* < 0.05), with average monthly increases of 346.166 yuan, 144.492 yuan, 78.814 yuan, 135.401 yuan, and 0.300, respectively. The instantaneous intervention effect (β_2_) analysis showed significant immediate declines in total cost, drug cost, examination fee, service fee, and cost efficiency index (*P* < 0.05), with reductions of 2,457.823 yuan, 1,400.017 yuan, 176.419 yuan, 799.948 yuan, and 0.212, respectively. However, no significant immediate changes were observed in length of stay, material cost, laboratory test cost, or time efficiency index (*P* > 0.05). The post-intervention trend (β1 + β3) indicated significant downward trends in length of stay, total cost, drug cost, material cost, service fee, time efficiency index, and cost efficiency index (*P* < 0.05), with average monthly decreases of 0.183 days, 438.788 yuan, 118.325 yuan, 151.734 yuan, 142.507 yuan, 0.028, and 0.038, respectively. In contrast, no significant downward trends were observed in examination or laboratory test costs (*P* > 0.05), highlighting the need for improved management in these areas.

Overall, the results in Tables 5, 6 demonstrate that the interventions for the GE13 and GE15 groups achieved significant success in reducing length of stay, total cost, and drug costs, particularly in controlling total and drug expenses. However, some indicators, such as material costs, examination fees, and laboratory test costs, did not show significant improvement, indicating a need for enhanced management strategies. Further measures should be implemented to optimize cost management and ensure comprehensive intervention effectiveness, such as establishing a DRG-based evaluation and approval system for materials, conducting cost-effectiveness analyses of materials, integrating material and diagnostic testing costs into standardized clinical pathways, reducing unnecessary examinations and tests, promoting mutual recognition of test results across institutions, and strengthening physician training and education. Additionally, shifting performance incentives from encouraging “more procedures” to rewarding “appropriate procedures” and “cost-saving practices” could help sustain these improvements.

## 4 Discussion and suggestions

### 4.1 Reasonably controlling drug and material costs and improving the performance evaluation system

Currently, hospitals implement a zero-markup policy for medications and medical supplies, making these items non-revenue-generating for medical institutions and key targets for cost control (7). Through rationalized management of medications, such as eliminating “free-riding” discharge medications and strengthening the management of antibiotics and adjuvant drugs, the medication costs for both the GE13 and GE15 groups have significantly decreased. However, the reduction in medical supply costs at the sample hospital was not significant, which may be attributed to the following reasons: (1) High dependency on medical materials: the GE1 disease group pertains to hernia repair surgery, for which the core specialized material is the hernia mesh patch. (2) Popularization of minimally invasive surgery: compared to traditional open surgery, laparoscopic procedures significantly increase the reliance on specialized instruments and consumables. (3) Complex cases and patient demands: for recurrent hernias and hernias with defects, surgeons are often compelled to select more expensive and reliable materials. (4) Patient preferences: patients with better economic conditions and higher quality-of-life expectations may insist on using expensive supplies. (5) Surgeons' usage habits. The lack of a significant decline in examination and laboratory test costs may be attributed to inadequate implementation of the mutual recognition system for test results, duplicate testing, physicians' overreliance on “evidence support,” and non-standardized medical practices.

The hospital operations management department should continue to regularly organize expert reviews on the rationality of medication and medical supply usage for key DRG groups and provide alternative lists to guide departments in selecting cost-effective options. Simultaneously, a comprehensive review of medical supplies in use should be conducted, focusing on both price control and rational usage management. By integrating indicators such as CMI and average length of stay, and fully considering professional characteristics, operational complexity, and technical risks, multi-departmental collaboration should be strengthened to establish and improve a supervision and evaluation mechanism aligned with DRG payment reform (8).

### 4.2 Shorter pre-operative hospital stay, reduces the average length of stay

A shorter average length of stay (ALOS) can reduce hospitalization time and medical costs for patients, improve the utilization efficiency of medical resources, and reflect enhanced specialized technical capabilities and improved quality control in disease management (9). For patients with a clear diagnosis and stable condition, specialists should actively encourage patients to arrange for pre-admission. After the implementation of the intervention measures, the number of pre-admissions steadily increased, shortening the average pre-operative hospital stay. In 2023, the average length of stay for groups GE13 and GE15 significantly decreased compared with that in 2022, while bed turnover rates improved, enhancing service efficiency and operational efficiency. Additionally, the reduction in the average length of stay also lowered costs such as bed fees and service fees, contributing to a decrease in overall hospitalization expenses.

However, the average length of stay is not simply shorter; rather, it should be minimized as much as possible while ensuring patient safety (10). Only by continuously improving medical technology, optimizing patient flow, and refining clinical pathways can healthcare institutions enhance efficiency and effectively reduce the average length of stay.

### 4.3 Achieving deep integration of DRGs and CPs via CPs to support DRG implementation

Clinical pathways not only provide patients with standardized medical services, ensuring the quality of care but also, have a positive effect on controlling length of stay, medication costs, and material expenses (11). The principle of DRG grouping ultimately aims to achieve “the same disease group, same quality, and same price” (12). Clinical pathways and DRGs share inherent similarities and converging goals, both aimed at saving medical resources and reducing healthcare costs.

Taking the GE1 disease group (inguinal and ventral hernia surgery) as an example, its clinical pathway includes the following components: applicable subjects, diagnostic criteria, treatment plan selection, standard length of stay, criteria for entering the pathway, pre-operative preparation time, discharge criteria, and analysis of variations and their causes (13). Each component specifies the diagnosis and procedures in detail, reducing the risk of necessary treatments being cut to control costs. During the revision of the hernia clinical pathway based on the DRG payment structure for the GE1 disease group, sample hospitals added relevant auxiliary examinations and standardized the use of antibiotics, hemostatic agents, and preferred centralized procurement patches. Relying on clinical pathways for regulatory oversight and cost control of disease types helps hospitals avoid losses under the DRG payment system.

### 4.4 Continuously implementing MDT-based DRG disease group management to increase operational efficiency

The DRG management MDT team at the sample hospital collaborates with clinical departments across the institution on the basis of routine departmental work. They utilize actual DRG payment data to conduct comprehensive and in-depth analyses of clinical practices, DRG operations, and evaluations of drugs and materials. This guidance helps clinicians continuously standardize their practices and promotes the ongoing improvement of core DRG indicators. Special working groups focused on operational analysis, clinical pathways, and performance evaluation hold weekly operational analysis meetings. These meetings address issues related to key disease types in areas such as clinical processes, medications, materials, examinations, and laboratory tests, emphasizing optimization and control. This approach creates reasonable pathways that increase efficiency and control costs while ensuring quality.

### 4.5 Strengthening control over abnormal expense cases and increasing supervision of medical irregularities by health insurance departments

For cases of severe DRG overruns, internal appeals should be initiated, with health insurance and assessment departments organizing experts to evaluate complex and critical cases, as well as those involving new medical technologies that reflect the hospital's public service nature, providing performance incentives accordingly. Reports should be submitted in accordance with the requirements of the special DRG payment exceptions in Nantong. Departments with low reimbursement rates exceeding 10% will be closely monitored to identify risks such as inadequate treatment and low admission standards. For disease groups lacking reasonable charging standards or DRG payment criteria, active communication with regulatory authorities should be pursued to seek the addition of new charging items and adjustments to the weights of the disease groups.

Under the influence of the DRG “payment ceiling” effect, healthcare institutions may excessively control costs, leading to issues such as inadequate treatment, unnecessary high coding, and fragmented hospitalizations (14). On the one hand, this can affect the quality of care and patient recovery; on the other hand, it may lead to excessively reduced lengths of stay and an increased proportion of low-reimbursement cases. Therefore, health insurance departments should guide healthcare institutions to shift their operational management concepts and establish a regulatory system compatible with DRG reforms. Through the use of information technology, such as intelligent control in health insurance, big data detection and analysis of DRGs, and case quality control systems, irregularities and policy risk points can be accurately identified (15). Additionally, improving the daily supervision system, which should include methods such as surprise inspections, routine checks, expert reviews, interhospital assessments, and annual performance evaluations, is essential.

Conducted within the context of China's DRG reform, this study demonstrates that reductions in length of hospital stay, total costs, and cost efficiency index were not achieved by cutting medical services or refusing patients, but rather through improved efficiency in resource utilization while maintaining the quality of care. This research provides direct evidence of the reform's effectiveness in cost containment, thereby reinforcing the credibility of the reform policy. It also offers a basis for higher-level authorities to adjust DRG payment standards and coordinate supporting policies.

This is a retrospective single-institution study where data accuracy relies on the completeness of medical records and the precision of coding. Potential coding inaccuracies or omissions may exist, and the findings may not be broadly representative. The analysis of a single DRG group primarily focuses on indicators such as costs and length of stay, while critical dimensions of treatment quality, such as cure rates and patient satisfaction, are not sufficiently covered. In future studies, we plan to collect data from multiple peer hospitals within the city on this DRG group and other groups, aiming to expand the sample size and research scope. This will further elucidate the impact of China's ongoing DRG payment reform on hospital operations, physicians' clinical practices, and patient experiences.

## Data Availability

The original contributions presented in the study are included in the article/supplementary material, further inquiries can be directed to the corresponding author.
